# Identification of serum biomarkers for necrotizing enterocolitis using aptamer-based proteomics

**DOI:** 10.3389/fped.2023.1184940

**Published:** 2023-05-31

**Authors:** Stephen Mackay, Lauren C. Frazer, Grace K. Bailey, Claire M. Miller, Qingqing Gong, Olivia N. Dewitt, Dhirendra K. Singh, Misty Good

**Affiliations:** ^1^ Division of Neonatal-Perinatal Medicine, Department of Pediatrics, The University of North Carolina at Chapel Hill, NC, United States; ^2^ Division of Newborn Medicine, Department of Pediatrics, Washington University School of Medicine, St. Louis, MO, United States

**Keywords:** necrotizing enterocolitis, prematurity, aptamer, SomaScan, serum, biomarker

## Abstract

**Introduction:**

Necrotizing enterocolitis (NEC) is a potentially fatal intestinal disease primarily affecting preterm infants. Early diagnosis of neonates with NEC is crucial to improving outcomes; however, traditional diagnostic tools remain inadequate. Biomarkers represent an opportunity to improve the speed and accuracy of diagnosis, but they are not routinely used in clinical practice.

**Methods:**

In this study, we utilized an aptamer-based proteomic discovery assay to identify new serum biomarkers of NEC. We compared levels of serum proteins in neonates with and without NEC and identified ten differentially expressed serum proteins between these groups.

**Results:**

We detected two proteins, C-C motif chemokine ligand 16 (CCL16) and immunoglobulin heavy constant alpha 1 and 2 heterodimer (IGHA1 IGHA2), that were significantly increased during NEC and eight that were significantly decreased. Generation of receiver operating characteristic (ROC) curves revealed that alpha-fetoprotein (AUC = 0.926), glucagon (AUC = 0.860), and IGHA1 IGHA2 (AUC = 0.826) were the proteins that best differentiated patients with and without NEC.

**Discussion:**

These findings indicate that further investigation into these serum proteins as a biomarker for NEC is warranted. In the future, laboratory tests incorporating these differentially expressed proteins may improve the ability of clinicians to diagnose infants with NEC rapidly and accurately.

## Graphical Abstract

Overview of the study. Figure made with Biorender.com. 
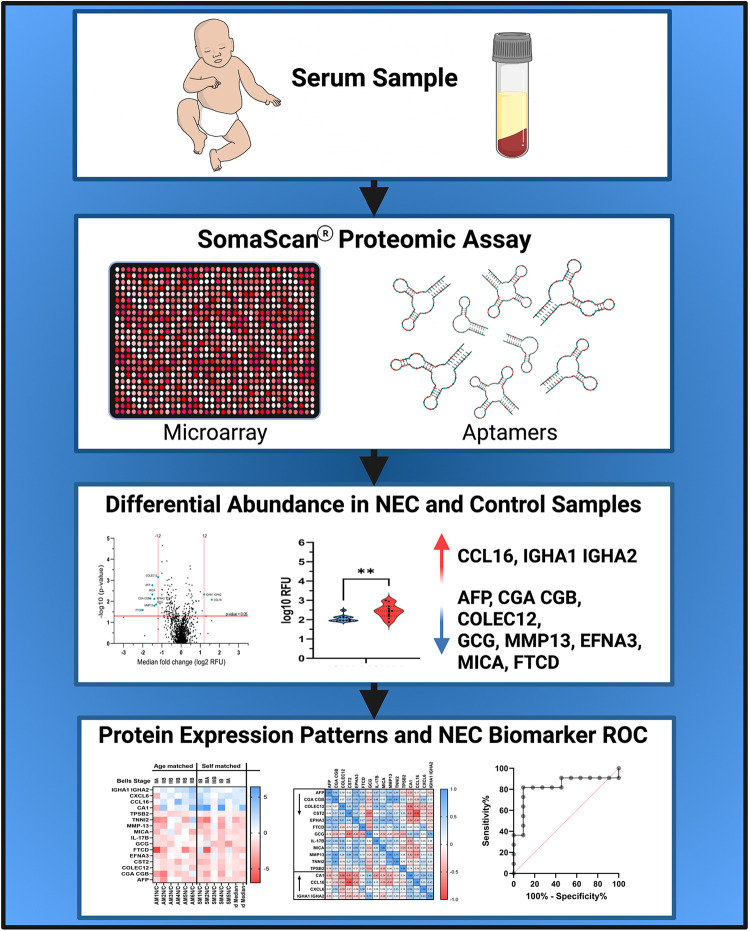

## Introduction

Premature and low birthweight infants are at risk for necrotizing enterocolitis (NEC), a severe inflammatory intestinal disease. The incidence of NEC is as high as 7% in preterm infants born at <32 weeks and 5%–22% in extremely low birth weight (ELBW, <1,000 g) infants (1). The symptoms of NEC are often nonspecific and subtle; however, neonates who develop NEC can rapidly worsen and progress to requiring emergency surgery or death within hours of diagnosis. Thus, accurately diagnosing NEC early in the disease course is crucial for initiating potentially life-saving clinical interventions (2). Unfortunately, diagnostic tools and treatment options for NEC have not improved despite decades of intensive research (3).

During NEC, intestinal barrier dysfunction resulting from epithelial injury and inadequate repair mechanisms can lead to bacterial translocation across the gut barrier, systemic inflammation, and potentially sepsis (4–8). Due to this systemic inflammatory response, symptoms of NEC can be nonspecific and difficult to distinguish from other disease processes. Identification of biomarkers for NEC that are both sensitive and specific would be a significant advance in clinical care and facilitate early diagnosis and treatment of neonates with NEC. Serum biomarkers for NEC are a potentially powerful tool that could rapidly differentiate infants with or without disease, but there are currently no effective predictive biomarkers routinely used in clinical practice.

Biomarkers for NEC have previously been investigated using liquid chromatography-tandem mass spectrometry (LC-MS/MS) (9) and enzyme-linked immunosorbent assay (ELISA) (10). This study uses an aptamer-based screening method to determine the relative expression of >1,300 protein targets (SomaScan®). This technology has been used to identify biomarkers in adult and pediatric diseases, including Duchenne's muscular dystrophy, ulcerative colitis, coronary heart disease, and cancer (11–21). Using this assay, we detected ten differentially expressed proteins in the serum of patients with and without NEC. This includes two that were upregulated and eight that were downregulated during NEC. ROC curves indicated that these proteins could effectively discriminate between patients with disease compared to those without. Future studies will focus on validating the efficacy of these potential NEC biomarkers in a larger patient population.

## Materials and methods

### Study design

In this prospective study, infants admitted to the St. Louis Children's Hospital Neonatal Intensive Care Unit (NICU) in St. Louis, Missouri, USA, were enrolled according to protocols approved by the Washington University in St. Louis School of Medicine Institutional Review Board (IRB protocol numbers 201706182 and 201802101)*.* Infants were eligible for enrollment if they were born between 22 and 42 weeks gestation and either developed NEC or were age-matched controls who did not develop NEC. Infants with any major congenital anomalies were excluded. Clinical information from the infant's medical record was collected from admission until discharge. For the present study, the cohort consisted of infants (*n* = 18) born between 24 and 36 weeks gestation diagnosed with NEC (*n* = 12) and age-matched controls (*n* = 6). Clinical information, demographic information, and NEC severity are summarized in Table 1. Modified Bell's Staging for NEC (7, 22, 23) was used to determine NEC severity.

**Table 1:** Description of patient cohort.

Infant data		Pregnancy and delivery details						Disease severity				
Sample ID	NEC (N) Control (C)	Gestational age	Sex	Birth weight (g)	Race (maternal)	Delivery route	Enrollment	NEC	Highest Bell’s stage	Surgical NEC	Radiographic Findings	Final disposition
**Age-matched**												
AM1N	N	34 0/7	Female	2360	White	CS	Case	Yes	IIA	No	PN	Discharged
AM1C	C	34 6/7	Female	1740	Black	CS	Control	No				Discharged
AM2N	N	26 0/7	Male	480	White	CS	Case	Yes	IIB	No	PN	Death
AM2C	C	33 4/7	Female	1940	Unknown	V	Control	No				Discharged
AM3N	N	36 2/7	Female	2330	White	CS	Case	Yes	IIB	No	PN, PVG	Discharged
AM3C	C	36 3/7	Male	3161	White	V	Control	No				Discharged
AM4N	N	24 5/7	Male	640	White	CS	Case	Yes	IIB	No	PN	Discharged
AM4C	C	26 3/7	Female	710	Black	CS	Control	No				Discharged
AM5N	N	31 0/7	Female	1190	White	CS	Case	Yes	IIB	No	PN, PVG	Discharged
AM5C	C	34 0/7	Male	2030	White	V	Control	No				Discharged
AM6N	N	24 2/7	Male	700	White	CS	Case	Yes	IIIB	PD, PR	#	Death
AM6C	C	27 0/7	Male	1150	White	V	Control	No				Discharged
**Self-matched**												
SM1N/C	C+N	26 0/7	Female	830	Black	CS	Control/Case	Yes	IB	No	N/A	Discharged
SM2N/C	C+N	28 6/7	Male	1220	Black	CS	Control/Case	Yes	IIIA	No	PN, PVG	Discharged
SM3N/C	C+N	25 4/7	Female	760	White	V	Control/Case	Yes	IIIB	PD	PN, PVG, P	Death
SM4N/C	C+N	25 1/7	Male	1620	Black	CS	Control/Case	Yes	IB	No	N/A	Discharged
SM5N/C	C+N	25 0/7	Male	760	Black	CS	Control/Case	Yes	IIA	No	PN	Discharged
SM6N/C*	C+N	24 4/7	Female	630	White	CS	Control/Case	Yes	IIIA	PD	PN, PVG	Death

SM6N/C* failed the assay quality control checks and was not included in the study analysis.

* N = NEC, C = Control, CS = cesarean section, V = Vaginal delivery, PR = Laparotomy, partial resection, PD = Peritoneal drain, PN = Pneumatosis, PVG = Portal Venous Gas, P = Pneumoperitoneum, # = gasless abdomen, bowel perforation, multiple surgeries for NEC. N/A = Not applicable

### Sample collection

Serum samples were collected once at enrollment for all participants (*n* = 18). A second serum sample was collected at the time of diagnosis if the infant developed NEC after enrollment (*n* = 6 self-matched infants). Age-matching was performed based on the post-menstrual age (PMA) of infants at the time of NEC diagnosis. There were six age-matched pairs included in this study (*n* = 12 infants). After collection, serum samples were centrifuged at 3,000 r.p.m. for 10 min, then subsequently aliquoted and stored at −80°C until analysis.

#### Proteomics assay

An aptamer-based SomaScan® (24) 1,300 serum protein microarray kit was used by the Genome Technology Access Center at the McDonnell Genome Institute at Washington University School of Medicine to identify biomarkers for each of the serum samples and respective controls according to the manufacturer's guidelines (SOMAlogic®, Boulder, CO, USA). Aptamers are 40 base pair oligonucleotides consisting of natural and modified nucleotides. These aptamers, called SOMAmers®, were immobilized on streptavidin beads. Proteins from serum were tagged with biotin, captured as a SOMAmer® reagent/protein pair, cleaved, denatured, and eluted prior to hybridization on a customized Agilent SureScan DNA microarray. We utilized a resolution of 5 µm and detected Cy3 fluorescence expressed as relative fluorescence units (RFU). Off-scanner raw signal values were calibrated, standardized, scaled at 40%, 1%, and 0.005%, and normalized. The RFU readout intensities are directly proportional to the amount of target protein, performed using Agilent Feature Extraction v10.7.3.1. Differential abundance was calculated using the SomaScan® statistical analysis tool v4.1 (SOMAlogic®) and subjected to a linear model fitting of the signal data and an empirical Bayesian statistical test for group comparisons. Samples were screened by row check intensity scaling and target biomarkers by column check quality control intensity scaling, where aberrant intensities are flagged for exclusion during data analysis. One self-matched sample pair (SM6N and SM6C) was excluded from further analysis due to aberrant scaling in the quality control row check. The raw data file is available in Supplementary Table S1.

#### Statistical analysis

RFU data was normalized by log transformation. Log2 transformations were used to generate volcano plots and heat maps for median and individual sample and target comparisons. A median fold change cut-off value of ≥1.2 and a *P*-value cut-off of ≤0.05 were used for differentially abundant biomarker selection based on the proteomics data using unmatched and matched sampling. Log2 median fold change transformations of significant proteins were used to create Pearson's correlation matrices and calculate the area under the curve (AUC) for Receiver Operating Characteristics (ROC). Confidence intervals of 95% were calculated by Wilson/Brown method. Log10 transformations, of case and control samples, were used to generate violin plots using the Tukey method and a paired parametric one tailed *t*-test. A *z*-score heat map was used to test for variation in case and control samples for each of the differentially abundant targets. All figures and statistics were generated using GraphPad Prism 9.3.1. Gene classification was standardized using the DAVID functional annotation tool (25).

## Results

The clinical characteristics of the infants in this study (*n* = 18) are summarized in Table 1. Patients (*n* = 6) enrolled at the time of NEC diagnosis were paired with age-matched (*n* = 6) controls based on post-menstrual age. In addition, patients that were enrolled in our prospective study as controls and subsequently developed NEC (*n* = 6) were grouped in a self-matched cohort. For the self-matched cohort, we analyzed protein levels in samples obtained at the time of enrollment and upon a diagnosis of NEC. One self-matched pair, patient SM6N/C, was excluded from further analysis due to failed quality control measures, as delineated in the methods section. Thus, 17 infants in total, including 12 in the age-matched and 5 in the self-matched cohort, were included in further analysis.

**Figure 1:**
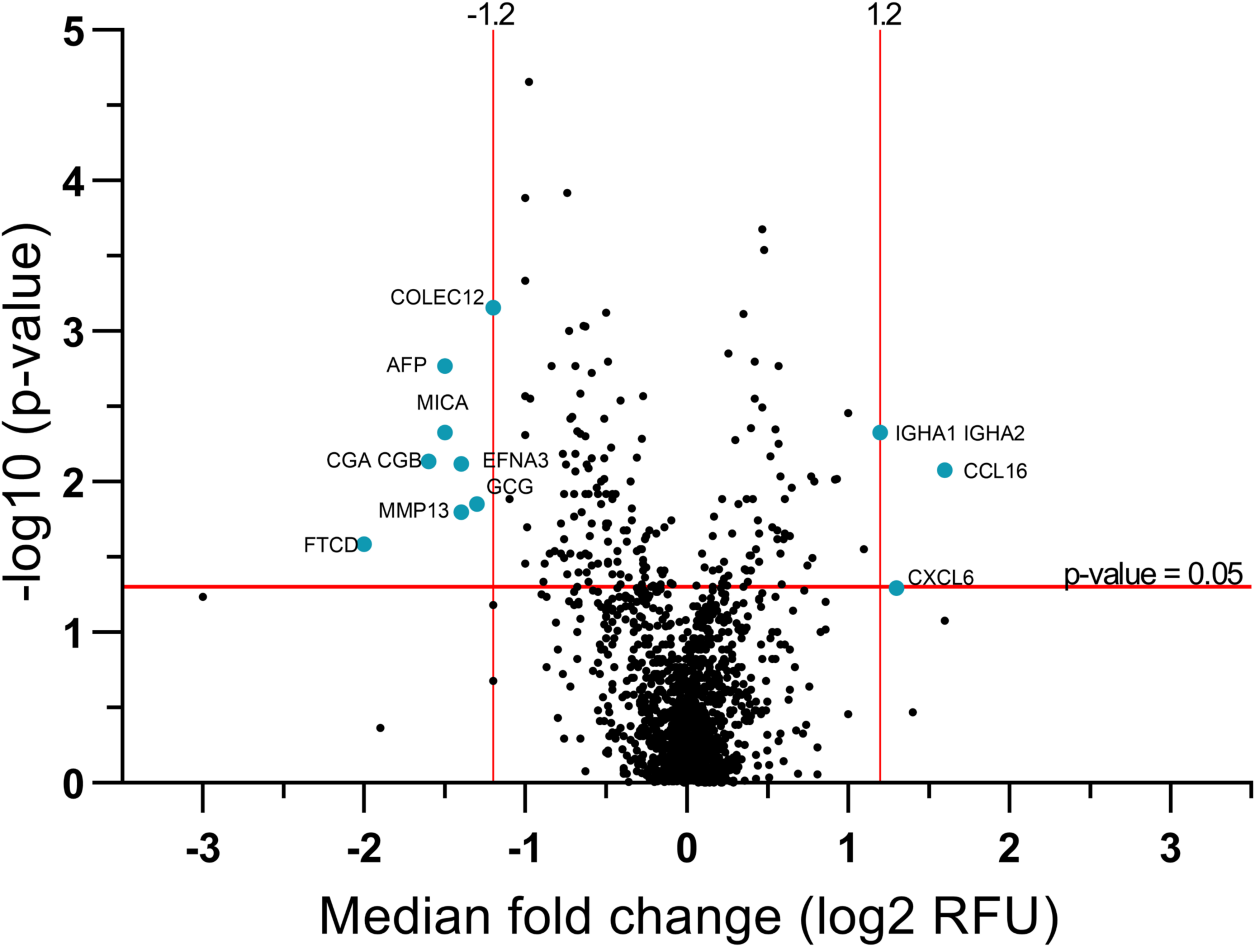
Volcano plot of the relative levels of serum proteins in patients with and without NEC identifies 10 differentially expressed proteins. Differential abundant proteins were selected and presented on a volcano plot based on median fold change (log2 RFU) and *P*-value (−log10 *t*-test). Statistically significant proteins of interest (blue dots) were selected based on median fold-change cut-off values (log2 ± 1.2) and *P* < 0.05 indicated as red lines. All selected proteins were statistically significant except CXCL6 (*P *= 0.051), which was considered potentially clinically significant.

In this study, we measured the relative abundance of over 1,300 serum proteins using an aptamer-based proteomic assay. Of the over 1,300 analyzed proteins, ten proteins (two increased and eight decreased in relative abundance) were significantly different between patients with NEC and controls (Figure 1). Proteins of interest were selected based on meeting the criteria of a median fold-change of ±1.2 and *P*-values <0.05 for the log transformed RFU data. Serum proteins that were increased during NEC included C-C motif chemokine ligand 16 (CCL16) and immunoglobulin heavy constant alpha 1 and 2 heterodimer (IGHA1 IGHA2). Proteins that were decreased during NEC included collectin subfamily member 12 (COLEC12), glucagon (GCG), alpha fetoprotein (AFP), formimidoyltransferase cyclodeaminase (FTCD), matrix metallopeptidase 13 (MMP13), glycoprotein hormone alpha polypeptide heterodimer (CGA CGB), MHC class I polypeptide-related sequence A (MICA), and Ephrin A3 (EFNA3). Differentially abundant protein biomarkers are summarized in Table 2.

**Table 2:** Details of differentially expressed proteins in the serum of neonates with NEC relative to controls.

Protein		Function	Tissue origin	Role in intestinal development, inflammation, and NEC	References
Increased in NEC vs. controls					
C-C motif chemokine 16	CCL16	Chemokine	Neonatal liver, macrophages, and lymphocytes	• Chemotactic toward monocytes and lymphocytes. • Induced expression by IL-10, LPS, and IFN-*γ* in activated monocytes and lymphocytes. • Ligand for CCR1, CCR2, CCR 5, and CCR8 cell surface and H4 eosinophil and mast cell receptors.	(26)
					(27)
					(28)
Immunoglobulin A	IGHA1 IGHA2	Mucosal antibody	Maternal milk in neonates	• IGHA1 IGHA2 is the mucosal specific heterodimer. • Decreased binding of IgA has been shown to correlate with intestinal dysbiosis.	(29)
					(30–32)
C-X-C motif chemokine 6	CXCL6	Chemokine	Macrophages	• IL17A induced chemokine for neutrophils. • Signal through CXCR5 and CXCR7 receptors.	(33)
					(34)
Decreased in NEC vs. controls					
Collectin-12	COLEC12	Scavenger receptor	Placenta, small intestine, and colon	• Involved in host defense promoting recognition and phagocytosis of Gram positive and negative bacteria, and yeast.	(35)
Pro-glucagon	GCG	Intestinal barrier development	Enteroendocrine cells	• Pro-glucagon cleaved into glucagon-like peptide-2 (GLP-2). • GLP-2 decreases enterocyte apoptosis, stimulates intestinal growth, crypt cell proliferation and villus formation. • GLP-2 promotes inflammatory cytokine production, delays NEC onset, and decreases mucosal barrier disruption.	(36)
					(37)
Ephrin-A3	EFNA3	Epithelial development	Small intestine and peripheral leukocytes	• GPI-anchored ligand of Eph receptors involved in signaling during migration and adhesion of epithelial development.	(38)
Collagenase 3	MMP13	Intestinal barrier function	Chondrocytes, connective and soft tissues	• Metalloprotease involved in the regulation of the intestinal barrier during inflammation by TNF signaling. • Reduced MMP-13 expression is a protective response to LPS induced inflammation. Involved in wound healing.	(39)
MHC class I polypeptide-related sequence A	MICA	Intestinal stress signaling	Gastric epithelium, endothelium, and monocytes	• MICA is specifically expressed in enterocytes as a stress induced-antigen recognized by intestinal epithelial γ*Δ* T-cells. • Over expression of MICA is associated with dysregulation of mucosal homeostasis.	(40)
Alpha-Fetoprotein	AFP	Plasma transport protein	Fetal liver	• Neonatal functional analog of serum albumin.	(41)
Human chorionic gonadotropin	CGA CGB	Developmental hormone	Placenta	• Heterodimer hormone. • Low CGA CGB expression is associated with poor development and low birth weight.	(42)
Formimidoyltransferase-cyclodeaminase	FTCD	Histidine metabolism	Fetal liver	• Functions as a transferase and a deaminase converting histidine to folate through the histidine degradation pathway. • Low histidine metabolism has been associated with NEC.	(43)

**Figure 2:**
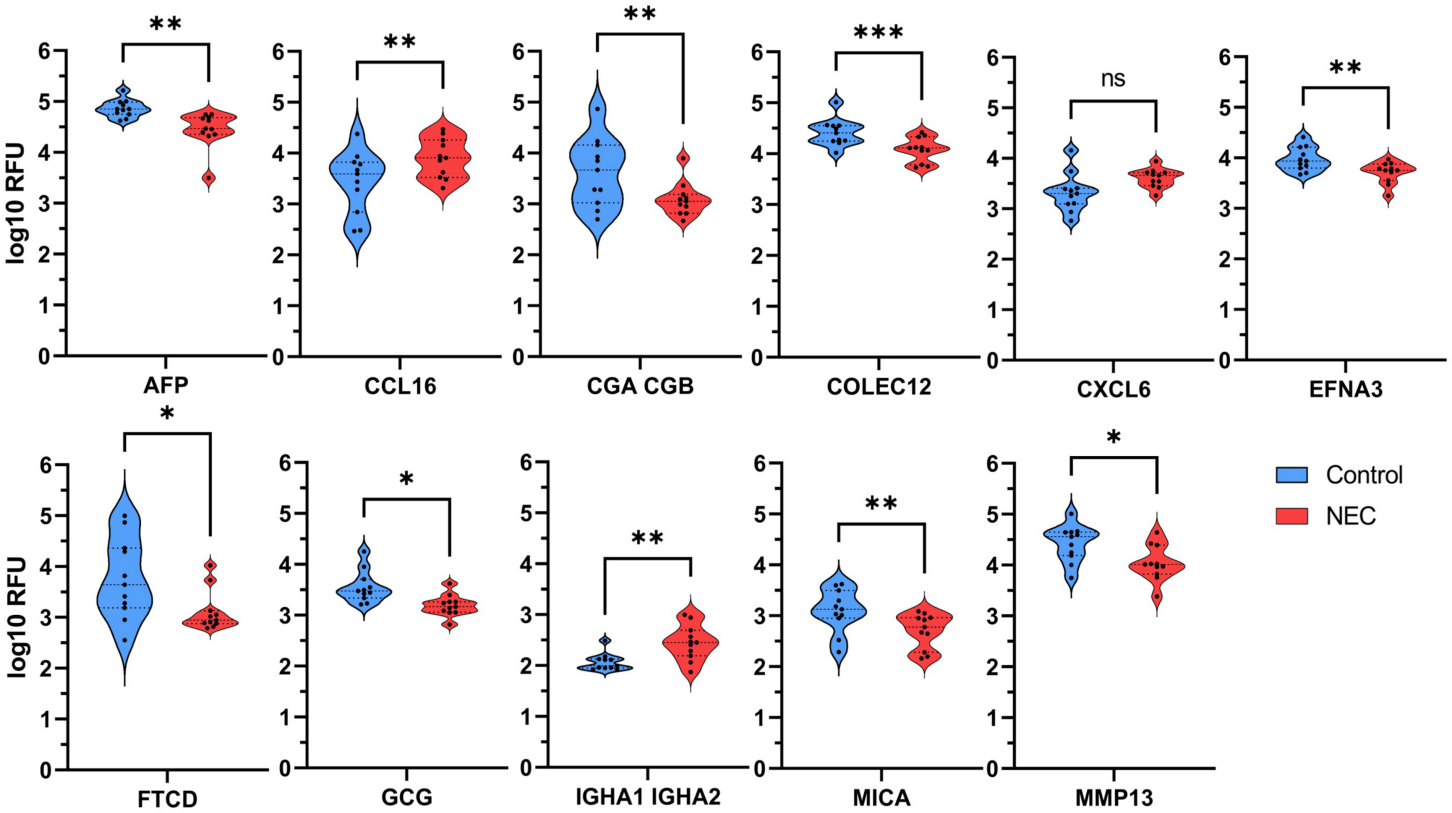
Significant differences in individual serum proteins were detected between patients with NEC and controls. Differentially abundant proteins were calculated for median distribution by log10 RFU. The median distribution is represented by the central dotted lines, and outer dotted lines indicate first and third quartiles. All selected proteins were statistically significant except CXCL6 (*P *= 0.051), which was considered potentially clinically significant. * *P *< 0.05, ** *P *< 0.01, *** *P *< 0.005 via a paired parametric two-tailed *t*-test. ns, not significant.

**Figure 3:**
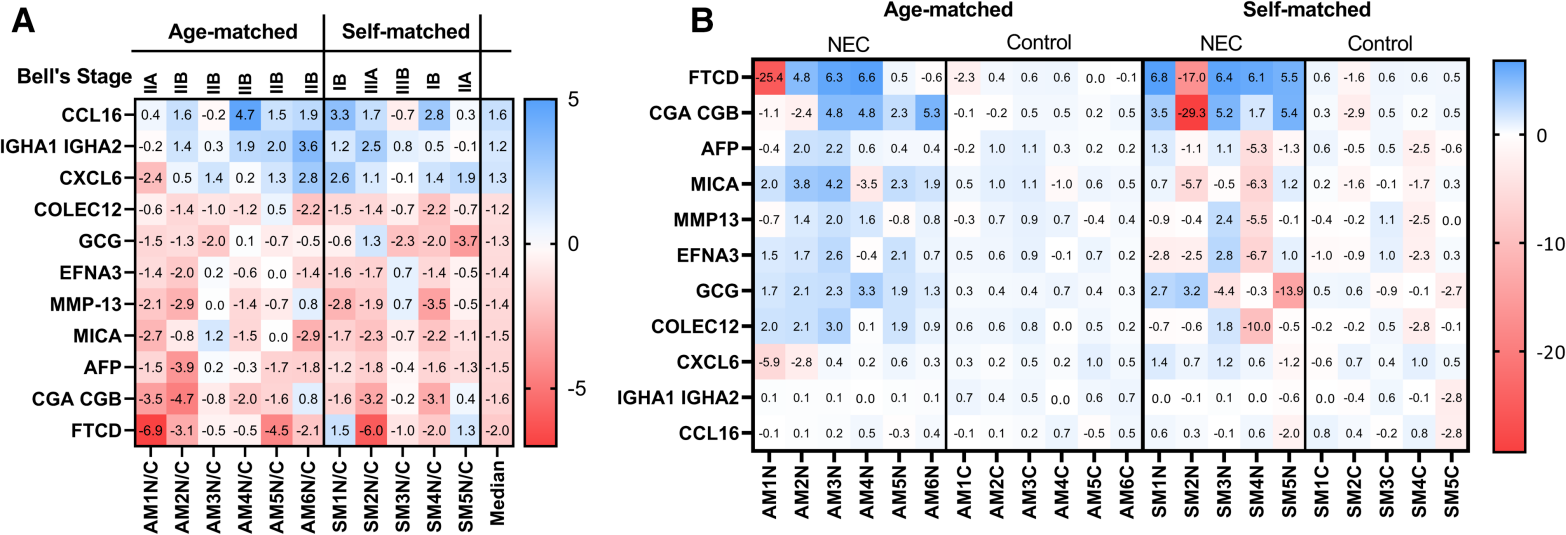
Similar relative abundance of serum proteins across the cohort when comparing matched NEC/control patient pairs. Heat maps of the differentially abundant proteins (**A**) in individual age-matched and self-matched pairs and (**B**) by *z*-score distribution of individual samples ranging between +6.78 to −29.3 where the sum of the *z*-score across protein targets = 1.

The relative abundance of the proteins of interest, as determined by Log10 transformations of the RFU values, was compared in serum samples from patients with NEC and controls. We found that all 10 proteins identified in Figure 1 were significantly different between these groups (*P* < 0.05, Figure 2). CXCL6 was not considered statistically significant (*P *= 0.051) but was included due to its potential clinical significance as an inflammatory protein. We next generated a heat map to provide a visual representation of the relative abundance of the proteins of interest across self- and age-matched pairs (Figure 3A). There was a remarkable degree of consistency in the patterns of protein expression across patient pairings. Using a heat map (Figure 3B), we observed that protein expression was similar across control samples and that the greatest variation in the matched pairs was present between patients with NEC.

**Figure 4:**
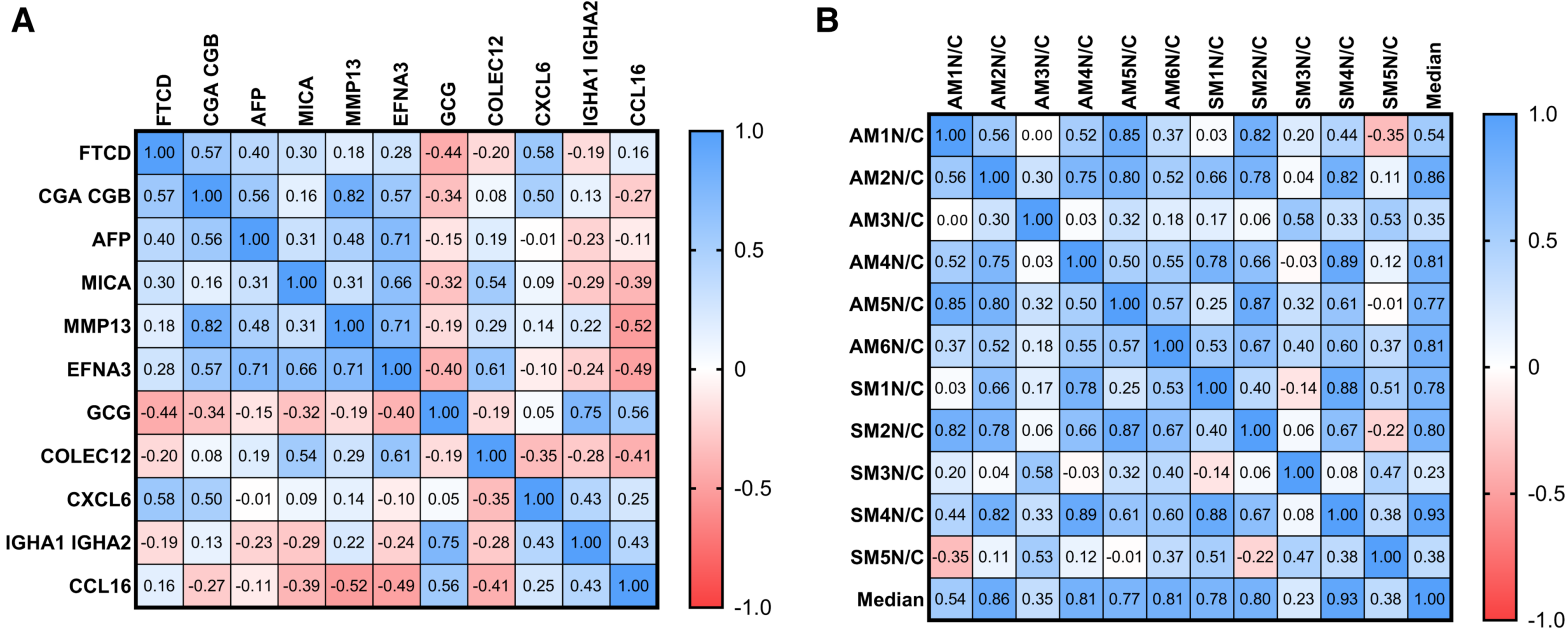
Pair-matched samples showed consistent correlations among sample pairs and individual protein biomarkers. Pearson's correlation matrixes between (**A**) serum proteins and (**B**) individual patient samples were calculated using median fold change (log2) in a parametric two-tailed test.

To determine if there was a statistical correlation between different protein levels, parametric two-tailed Pearson's correlation matrixes were generated. We found that the proteins increased in samples from patients with NEC shared positive correlations with each other and an inverse correlation with proteins of decreased abundance in patients with NEC (Figure 4A). All samples correlated positively with the median (Figure 4B) except sample pairs AM1N/C-SM5N/C, SM5N/C-SM2N/C, and SM1N/C-SM3N/C. These sample pairs had significant variations for the following specific proteins (Figure 3A); AM1N/C-SM5N/C for FTCD and CXCL6; SN5N/C-SM2N/C for FTCD and GCG, SM1N/C-SM3/NC for FTCD and CCL16.

**Figure 5:**
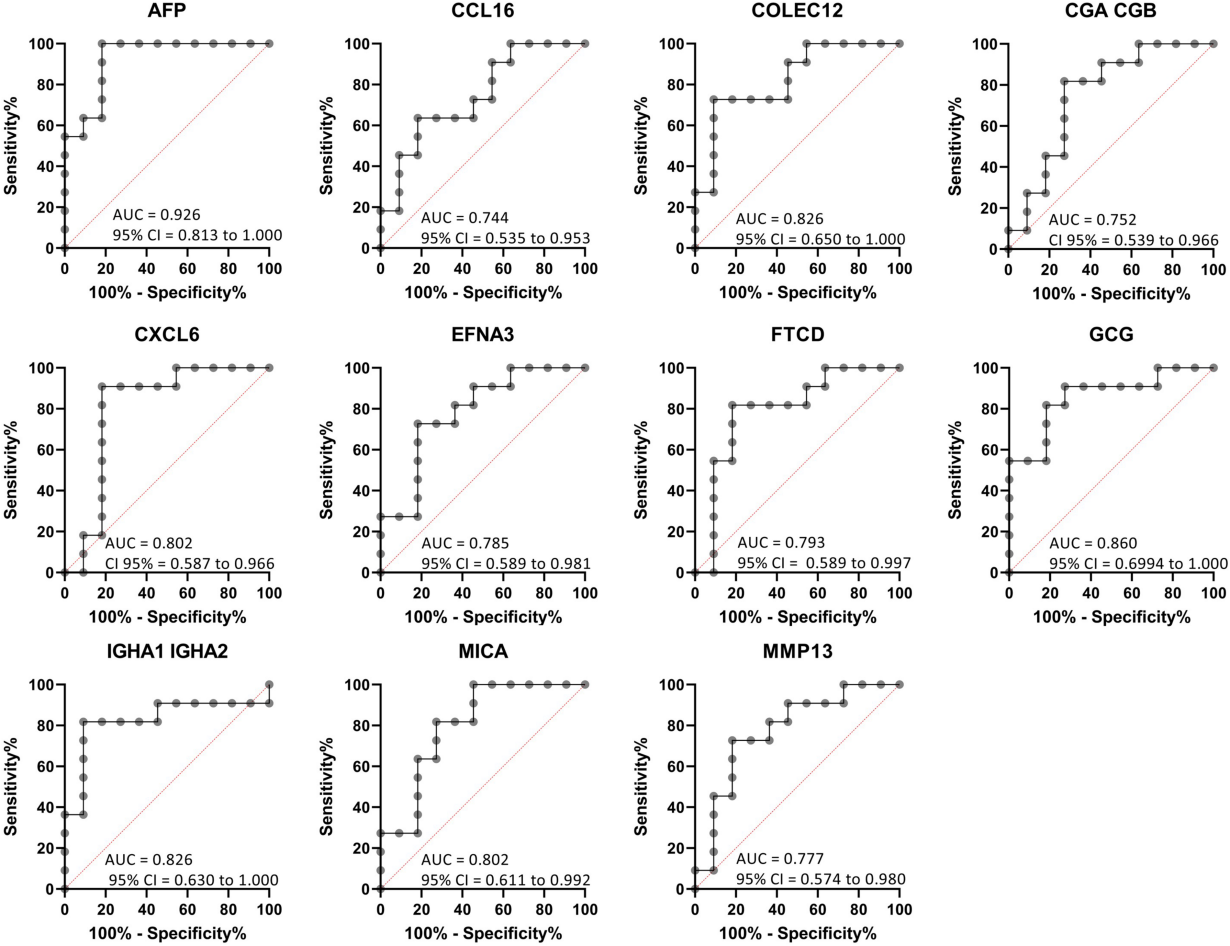
Receiver operating curves (ROC) of target proteins indicate that the differentially expressed serum proteins effectively discriminate between patients with and without NEC. Differentially abundant proteins (*n* = 10) were screened for sensitivity vs. specificity based on median log10 transformed RFU data for matched NEC and control samples. ROC curves were calculated using the Wilson/Brown method with a confidence interval (CI) of 95%. AUC values >0.7 are considered valid diagnostic biomarkers.

Individual biomarker sensitivity vs. specificity for identifying patients with NEC was measured by Receiver Operator Characteristic (ROC) curves and area under the curve (AUC) (Figure 5). ROC curves generated from combined self- and age-matched pairs identify values for given biomarkers where true positive (sensitivity) and percentage of true negative (100% specificity) are the most effective. An AUC value approaching 1 is a perfect diagnostic test. AUC values above 0.7 are considered acceptable, while AUC values above 0.8 are considered good for diagnostic tests. The AUC for proteins increased in the serum of patients with NEC relative to controls were as follows: CCL16 (AUC = 0.744, 95% CI = 0.535–0.953), CXCL6 (AUC = 0.802, 95% CI = 0.587–0.966) and IGHA1 IGHA2 (AUC = 0.826, 95% CI = 0.630–1.00). The AUC for proteins decreased in the serum of patients with NEC relative to controls were: AFP (AUC = 0.926, 95% CI = 0.813–1.00), MMP13 (AUC = 0.777, 95% CI = 0.574–0.980), FTCD (AUC = 0.793, 95% CI = 0.589–0.997), MICA (AUC = 0.802, 95% CI = 0.611–0.992), EFNA3 (AUC = 0.785, 95% CI = 0.589–0.981), GCG (AUC = 0.860, 95% CI = 0.6994–1.00), COLEC12 (AUC = 0.826, 95% CI = 0.650–1.00), and CGA CGB (AUC = 0.752, 95% CI = 0.539–0.966). These values demonstrate high sensitivity vs. specificity for all biomarkers of interest.

## Discussion

NEC is a rapidly progressive disease that can be difficult to diagnose using currently available tools. Identification of highly sensitive and specific biomarkers would allow for earlier initiation of potentially lifesaving treatments for neonates with NEC. In this study, we utilized an aptamer-based approach to identify serum proteins that were differentially abundant in samples from infants with NEC relative to controls. Serum proteins that were significantly different between the groups are described in Table 2.

Two proteins were upregulated in the serum of patients with NEC compared to controls, CCL16 and IGHA1 IGHA2. CCL16 is a chemokine produced primarily in the liver and secreted into the blood (27). Its production is induced in monocytes by the cytokines interleukin (IL)-10 and interferon-gamma (IFN-γ) as well as by lipopolysaccharide (LPS) expressed by Gram-negative bacteria (27, 28). CCL16 has been shown to induce lymphocyte and monocyte chemotaxis (26). Increased levels of CCL16 in the serum of neonates with NEC may reflect the inflammatory milieu of the intestine, which would support its use as a biomarker of NEC.

Serum IgA is monomeric (∼90% IGHA1, 10% IGHA2), whereas IgA derived from maternal milk and present in the intestinal mucosa in the form of secretory IgA (s-IgA), is typically a heterodimer of IGHA1 IGHA2 (44). In infants, IgA is derived solely from maternal milk for the first four weeks of life, until B-lymphocytes populate the intestine (30). The increased abundance of IGHA1 IGHA2 in the serum from neonates with NEC may be reflective of the gut barrier dysfunction observed during NEC (45), which would result in increased circulating levels of this primarily intestinal antibody. The level of IgA bound to the Gram-negative *Enterobacteriaceae* in the stool of preterm neonates is inversely correlated with the risk of NEC (30); however, how serum levels of IGHA1 IGHA2 correlate with NEC has not been previously explored.

We also detected eight proteins that were decreased in the serum of patients with NEC relative to controls. The two proteins that were the most effective at discriminating between patients with and without NEC included AFP (AUC = 0.926) and GCG (AUC = 0.860). AFP is elevated in preterm infants (<37 weeks) and normally decreases rapidly after birth (by 50% in the first 5 days of life in term infants) (41). AFP has been associated with the downregulation of inflammation (46); thus, decreased levels may contribute to the exaggerated inflammatory response observed in neonates with NEC.

GCG regulates blood glucose levels by promoting gluconeogenesis and glycogenolysis. Pro-glucagon is cleaved into several peptides involved in glucose metabolism and gastric function. Importantly, one of the peptide products, glucagon-like peptide 2 (GLP-2), decreases enterocyte apoptosis and stimulates intestinal growth, crypt cell proliferation, and villus formation (36). GLP-2 was also shown to have a protective and anti-inflammatory role in a rat model of NEC (37). It is possible that reduced levels of GCG found in the serum of patients with NEC may indicate that decreased GCG-mediated intestinal protection was associated with increased intestinal injury.

To our knowledge, this is the first study to analyze serum proteins using an aptamer-based assay on samples derived from infants with or without NEC. The traditional technique for differential analysis and quantitative proteomics is liquid chromatography coupled with mass spectrometry (LC-MS/MS). However, improvements in affinity capture and quantitation methods have allowed for alternative methods, which can address biases and limitations in LC-MS/MS and other platforms. A comparative analysis of LC-MS/MS, RNA sequencing, and SomaScan® analysis of mesenchymal and human embryonic stem cells showed a greater identification of unique markers using SomaScan® than LC-MS/MS and RNA sequencing. The benefits of this aptamer-based technology include a high dynamic range, low sample requirements (20 µg protein to 50 µl serum), and high sensitivity with improved detection of small molecule targets (47). This improvement in technology facilitated our detection of new potential serum biomarkers for NEC.

Limitations of this study included the relatively small sample size, the inability to match patients based on factors other than age, and the inclusion of two patients with Stage 1B NEC. This is a pilot study that will be expanded upon in future studies involving larger patient cohorts, which will allow for more detailed matching of patient characteristics and stratification of patients based on disease severity.

Studies analyzing serum biomarkers in preterm infants are complicated by several factors, including limited sample volumes, inflammatory proteins not specific for NEC, and age-specific changes in protein levels (48). We attempted to overcome these challenges by using an assay with high sensitivity, which allowed the detection of protein levels with a small volume of blood. In addition, we utilized age matching to limit confounding in our comparison of serum protein levels. We also found similar patterns of protein abundance in the age-matched and self-matched cohorts, which pointed to differences in protein levels being related to NEC and not post-menstrual age in the self-matched group. Finally, this study employed an unbiased screening approach, which provided the highest likelihood of identifying new biomarkers for NEC.

In conclusion, serum protein levels from infants with NEC were compared to controls using an aptamer-based proteomic assay with the successful identification of 10 proteins that were able to differentiate between the groups. Future studies will focus on the validation of these results in a larger patient cohort. The overarching goal is to improve the speed and accuracy in which clinicians can diagnose NEC to improve outcomes for critically ill neonates.

## Data Availability

The original data presented in the study are included in the article/**Supplementary Material**, further inquiries can be directed to the corresponding author.
